# Care and compassion at time of pandemic: an ICU field experience in Italy

**DOI:** 10.1186/s44158-021-00031-6

**Published:** 2022-01-12

**Authors:** Annalaura Ferrari, Selena Russo, Catia Quagliotto, Roberta Granello, Lorenza Menato, Antonio Nola, Stefano Addesa, Sergio Cassella, Antonio Farnia, Mariagrazia Strepparava, Alessandra Mauri, Mario Peta

**Affiliations:** 1 Department Hospital Medical Direction, Health Psychology Unit, AULSS 2 Marca Trevigiana, Treviso Hospital, Treviso, Italy; 2 Department of Medicine and Surgery, University of Milano – Bicocca, Milan, Italy; 3 Department of Anaesthesia and Intensive Care Units, AULSS 2 Marca Trevigiana, Treviso Hospital, Treviso, Italy; 4 Department of Mental Health, ASST Monza, Monza, Italy

**Keywords:** COVID-19, Pandemics, Intensive care units, Family visits, Italy, Healthcare workers’ well-being, Family-centred care

## Abstract

After the COVID-19 pandemic outbreak in March 2020, the majority of hospital policies have followed guidelines aimed to prevent the virus transmission and the families’ entry was denied in all hospital wards and intensive care units (ICU). Despite being necessary, such restrictions have been experienced with discomfort and sufferings by the general ICU staff of Treviso Hospital (Italy) and by families of patients. Therefore, from April 2020, a step-by-step project was developed to reactivate contact with COVID-19 patients’ families. The several requests and appeals of intensive care communities and organizations, both nationally and internationally, motivated the Treviso hospital initiative, leading to a model that might be potentially useful to other intensive care units worldwide.

## Background

When the COVID-19 pandemic first hit Italy in March 2020, relatives of patients were prevented from entering hospital wards, interrupting a decades-long effort to promote visitor access and foster a family-centred approach to intensive care unit (ICU) care [1–3]. Several researches are pointing out the negative impact of restricted visitation policies, in acute care settings, on the mental health and psychological well-being of patients, families, and healthcare workers (HCW) [4–6]. As the course of the pandemic progressed, scientific societies have initiated a debate about restrictions’ implications and responsive guidelines to allow hospital visitations. Existing studies and recommendations suggest that, if properly conducted, visits to COVID-19 or other hospital wards, do not endanger the safety of patients, visitors and HCW [1, 3]. Furthermore, Mistraletti et colleagues’ viewpoint highlights that, even if limited in time and conditioned by personal protective equipment (PPE) usage, family visits remain a fundamental necessity for families, patients and the whole ICU community [1]. Motivated by these findings, the Treviso Hospital devised a protocol to reopen the General ICU to families during the COVID-19 pandemic.

The general ICU of the Hospital of Treviso is located in one of the first and most affected Italian regions hit by the COVID-19 pandemic. Between May 2020 and June 2021, 360 patients, with acute respiratory failure due to COVID-19 infection, were admitted and treated. Since visitors’ access was suspended, the ICU team, with their strong attention and commitment to the communicative and relational aspects of care, firmly demanded to resume contact with families. With this towering aim, the ICU team’s endeavour has led to a progressive step-by-step project pointing to reopen the ICU to family members, so that a patient- and family-centred high-quality care could be restored.

## Phase one: letters to families

**Fig. 1:**
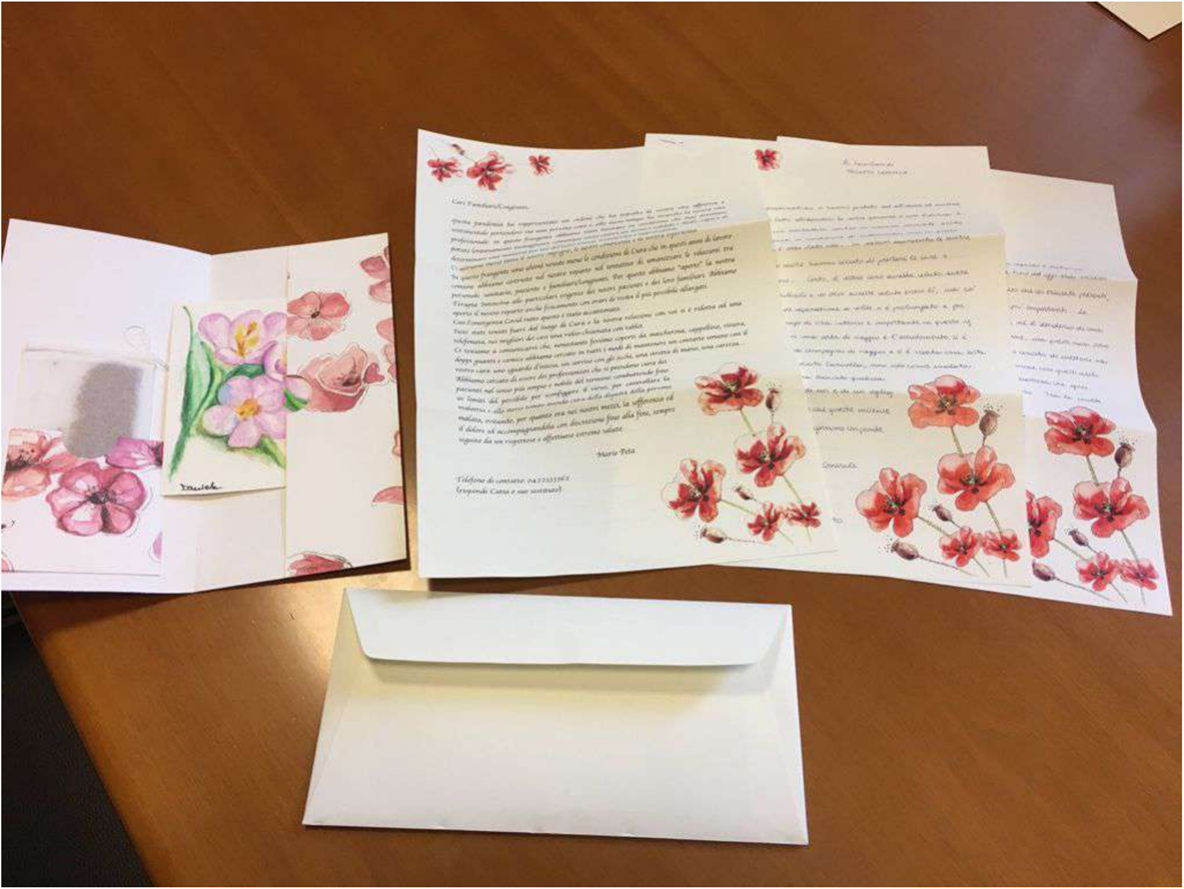
An example of a letter sended to the families

From March to April 2020 more than 30 COVID-19 patients died isolated from their families. ICU workers felt compelled to get in touch with patients’ families to make up for the ‘loneliness’ and the lack of the last farewell for their beloved one that families had to face. In April 2020 a narrative medicine project was implemented with the assistance of the Emergency Psychological Unit. On the spontaneous initiative of the ICU staff, an “end of life narrative” for each deceased patient was collected amongst HCW and transformed into a personalised story-letter that was sent to the patient’s family, after consent was obtained. The verbal consent to receive the letter was obtained through a telephone call. The letters had a common introduction and a personalised section recalling patients’ end-of-life moments and ICU staff’s messages for families, reassuring them that their loved ones had been cared for with humanity and that ‘they did not die alone’. Twenty-eight handwritten letters were sent to families accompanied by watercolour drawings and some plant seeds, metaphor of hope and rebirth (Fig. 1). After receiving the letter, all the families contacted by ICU staff expressed their appreciation in different ways, some came to collect the letter by themselves at the Hospital, some called back to thank and some wrote a thank you note to the staff. The ICU staff, during the follow-up meetings with the psychologist that followed the entire project, described the experience of phase-one as emotionally intense and extremely rewarding. The families’ positive responses suggest that the narrative medicine project succeeded in establishing contact between ICU HCW and patients’ families and supporting families’ mourning process. Concurrently, the project helped ICU HCW to express and process their experience and emotions linked to the pandemic period.

## Phase two: access to the morgue

In November 2020, a morgue room was set to let family members meet their departed relatives. Access to the COVID-19-area of the morgue was regulated by rigid rules and procedures relating to the infection containment, which were described in an official protocol approved by the Legal Office of the Hospital.

## Phase three: families’ visits

**Fig. 2:**
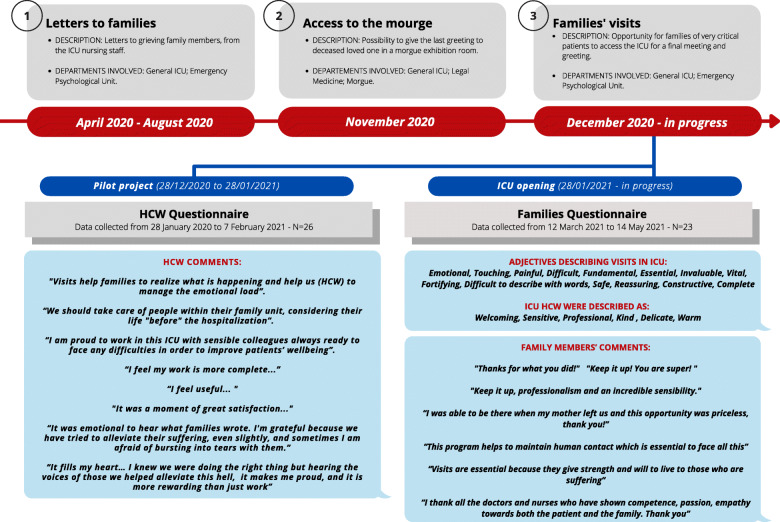
Project timeline and surveys. Project timeline; the different departments involved; some of HCW and family members’ comments, collected through the questionnaires. Abbreviations: *ICU*, intensive care unit; *HCW*, healthcare workers

From December 2020 to January 2021, a pilot project was implemented to allow families’ visits for ICU patients in end of life condition. Clinical and logistic barriers to families’ access to ICU [1] were analysed and a protocol duly describing the procedures for entrance was developed. To monitor ICU HCW’s experience and gather families’ feedback, both stakeholders involved in pilot study Phase 3 were invited to fill in an online survey. In Fig. 2 some of HCW and family members’ comments. During the pilot study, practical and emotional aspects of families’ visits were considered and discussed by ICU HCW in regular meetings with the staff of the Emergency Psychological Unit. Families’ gratitude and positive feedback enhanced a sense of professional and emotional competence and satisfaction with the project in ICU HCW.

In light of the positive feedback from families and the perceived quality by ICU HCW, reported in the online surveys (Fig. 2), at the end of January 2021 the ICU families’ visits project was broadened to include visitors of less severe patients, which means patients with non-invasive ventilation or in intubation. Between January and May 2021, a total of 70 families accessed the ICU of the Hospital of Treviso. It is important to state that there have been no cases of infection among the healthcare staff, nor among the patients’ family members, that could have occurred during the visits in the ICU.

## Conclusions

This project echoes and strengthens Mistraletti et al.’s [1] recommendations and the wider ICU community claim that ICU family visits during the pandemic and healthcare emergencies are feasible and most needed. Responses to the online surveys (Fig. 2) highlight that ICU visits not only are beneficial for patients and their families but they make HCW feel more competent and increase work wellbeing and motivation. Treviso Hospital experience, together with others researches, are offered in the hope to be useful to the international community to offer a foundation for the future development of interventions and protocols, targeted at a better integration of family visits in ICU care during pandemics and other health emergencies.

## Data Availability

Not applicable.

## Abbreviations

ICU: Intensive care unit
HCW: Healthcare workers
PPE: Personal protective equipment
